# Association of biomarkers with successful ventilatory weaning in COVID-19 patients: an observational study

**DOI:** 10.62675/2965-2774.20240158-en

**Published:** 2024-03-25

**Authors:** Bruna Schneider, Raquel Almeida de Oliveira, Gilberto Friedman, Rafael Barberena Moraes

**Affiliations:** 1 Universidade Federal do Rio Grande do Sul Porto Alegre RS Brazil Postgraduate Program in Pneumological Sciences, Universidade Federal do Rio Grande do Sul - Porto Alegre (RS), Brazil.; 2 Universidade Federal do Rio Grande do Sul Porto Alegre RS Brazil Universidade Federal do Rio Grande do Sul - Porto Alegre (RS), Brazil.

**Keywords:** Biomarkers, COVID-19, Coronavirus infections, Airway extubation, Intubation, intratracheal, Respiration, artificial, Ventilator weaning, Intensive care units

## Abstract

**Objective:**

To evaluate the association of biomarkers with successful ventilatory weaning in COVID-19 patients.

**Methods:**

An observational, retrospective, and single-center study was conducted between March 2020 and April 2021. C-reactive protein, total lymphocytes, and the neutrophil/lymphocyte ratio were evaluated during attrition and extubation, and the variation in these biomarker values was measured. The primary outcome was successful extubation. ROC curves were drawn to find the best cutoff points for the biomarkers based on sensitivity and specificity. Statistical analysis was performed using logistic regression.

**Results:**

Of the 2,377 patients admitted to the intensive care unit, 458 were included in the analysis, 356 in the Successful Weaning Group and 102 in the Failure Group. The cutoff points found from the ROC curves were −62.4% for C-reactive protein, +45.7% for total lymphocytes, and −32.9% for neutrophil/lymphocyte ratio. These points were significantly associated with greater extubation success. In the multivariate analysis, only C-reactive protein variation remained statistically significant (OR 2.6; 95%CI 1.51 – 4.5; p < 0.001).

**Conclusion:**

In this study, a decrease in C-reactive protein levels was associated with successful extubation in COVID-19 patients. Total lymphocytes and the neutrophil/lymphocyte ratio did not maintain the association after multivariate analysis. However, a decrease in C-reactive protein levels should not be used as a sole variable to identify COVID-19 patients suitable for weaning; as in our study, the area under the ROC curve demonstrated poor accuracy in discriminating extubation outcomes, with low sensitivity and specificity.

## INTRODUCTION

Ventilatory weaning is a constant challenge for intensivists and has become even more complex during the severe acute respiratory syndrome coronavirus 2 (SARS-CoV-2) pandemic. Severe hypoxemia, use of corticosteroids, prolonged mechanical ventilation (MV), sedation and immobilization for long periods, increased ventilatory demand, variable disease course, work overload, and lack of resources are factors that are faced.^(1-4)^

Despite the adoption of protocols and adherence to guideline recommendations, the extubation failure rates remain at approximately 15 – 30%.^(5,6)^ In coronavirus disease 2019 (COVID-19) patients, this rate is approximately three times higher than that in non-COVID-19 patients.^(7)^ Extubation failure is associated with increased hospital mortality, prolonged intensive care unit (ICU) stay, and increased need for tracheostomy.^(8,9)^ However, unnecessary prolongation of MV is also associated with an increased risk of infection and mortality in the ICU.^(5,10,11)^ In this context, attempting to identify factors that assist in decision-making about the ideal time for discontinuation of mechanical ventilation becomes necessary.

Biomarkers, such as C-reactive protein (CRP), procalcitonin (PCT), and the neutrophil-to-lymphocyte ratio (NLR), have been widely used in bacterial infections, playing a diagnostic, prognostic, risk stratification, and antibiotic duration-defining role, despite their limited sensitivity and specificity.^(12-16)^ Lymphocytes also play a crucial role in the inflammatory response and balance of the immune system, and lymphopenia, a common finding in SARS-CoV-2 infection, is significantly associated with worse outcomes.^(17-19)^ In general, as inflammatory disease progresses, the lymphocyte counts decrease, and neutrophil counts increase. The NLR, a tool easily calculated from the complete blood count, is an indicator of systemic inflammation and an independent risk factor for poor prognosis.^(20,21)^ To date, few studies have evaluated the association between these biomarkers in COVID-19 patients and worse clinical outcomes, but none have evaluated their relationship with weaning.^(18,22-24)^

The objective of this study was to evaluate the association of biomarkers CRP, total lymphocytes, and the NLR with successful extubation of patients with ventilatory failure secondary to SARS-CoV-2.

## METHODS

### Study design and population

This was a retrospective, observational, and single-center study conducted on patients admitted to the ICU of the *Hospital de Clínicas de Porto Alegre* between March 2020 and April 2021. Patients included in the study met the following criteria: confirmed diagnosis of SARS-CoV-2 infection through molecular biology or rapid antigen test, aged 18 years or older, and undergoing mechanical ventilation for severe acute respiratory syndrome. The exclusion criteria included SARS-CoV-2 infection occurring after the need for mechanical ventilation, use of extracorporeal membrane oxygenation (ECMO), death or transfer to another hospital without extubation being performed, use of interleukin-6 inhibitors, absence of collection of analyzed biomarkers, and refusal to share data through the General Data Protection Law (LGPD - *Lei Geral de Proteção de Dados*), which is in force in Brazil.

The study was approved by the Ethics and Research Committee (CEP) of the institution (CAAE 40843120.4.0000.5327). The authors signed a Data Use Commitment Agreement, confirming their commitment to the anonymous use of the data. Due to the anonymization of patients and the absence of diagnostic or therapeutic interventions, the Free and Informed Consent Form was waived. The results were reported according to the STROBE guidelines (Strengthening the Reporting of Observational Studies in Epidemiology).

### Data collection

The following data were collected through chart review: general patient characteristics, such as sex, age, comorbidities, body mass index (BMI), and Simplified Acute Physiology Score 3 (SAPS 3) score; date of admission, hospital discharge, or death; date of intubation and extubation; use of corticosteroids; performance of tracheostomy; and biomarkers (C-reactive protein, total lymphocytes, and neutrophil-lymphocyte ratio) collected up to 24 hours before or after the time of intubation and extubation. From these laboratory data, variations in the levels of these biomarkers between the date of intubation and the date of first extubation were calculated. Potential confounding factors were selected a priori based on clinical knowledge and previous literature.

### Outcomes and definitions

In this study, we compared biomarker kinetics between the Extubation Failure and Success Groups. The primary outcome was extubation success, defined as the absence of reintubation or return to mechanical ventilation after 48 hours. Failure was considered the need to return to mechanical ventilation in less than 48 hours.

As secondary outcomes, we evaluated the time on mechanical ventilation until extubation, length of hospital stay, ICU mortality, hospital mortality, and tracheostomy use. The criteria for extubation were evaluated and decided by the bedside team, following institutional protocols, without the researchers’ influence. Hospital transfer outcomes were included due to the pandemic, with patients being transferred to lower complexity hospitals due to high resource demand. The parameter ‘tracheostomy preextubation’ refers to the performance of a tracheostomy during the same hospitalization without prior extubation. This practice is uncommon outside the context of a pandemic; however, during the pandemic, it was employed for some patients experiencing difficult and prolonged weaning, at least in our center. In these tracheostomized patients, extubation success was considered the maintenance of mechanical ventilation independence for at least 48 hours.

### Statistical analysis

The sample was considered from the total list of hospitalizations in the period. Categorical variables were reported as a percentage, and continuous variables were reported as the mean ± standard deviation (SD) or median (interquartile range [IQR]). Receiver operating characteristic (ROC) curves were drawn to find the best cutoff point for the biomarkers based on the best sensitivity and specificity. Associations were made with the logistic regression model, except for the variable "use of corticosteroids," in which Fisher's exact test was used since, in one of the groups, no patient used corticosteroids. A nonlinear association was detected involving age, time on mechanical ventilation until extubation, ICU time, and hospital time with extubation success, and a spline transformation was applied.

For multivariable analysis, the following adjustment variables were used: previous heart disease, preextubation tracheostomy, and time on mechanical ventilation until extubation, defined based on statistical significance, as described in table 1 (p < 0.05). The other variables with significance in table 1 were not used due to collinearity with those already mentioned. The results of the uni- and multivariable analyses were expressed in odds ratios (ORs) and respective 95% confidence intervals (95%CIs). In all analyses, a p value of < 0.05 was adopted as the level for statistical significance. The software used was PASW Statistics for Windows version 18.0 and R version 4.2.0.^(25,26)^

**Table 1:** Characteristics of the population

Characteristics		Extubation failure (n = 102)	Extubation success (n = 356)	p value
Age		55 ± 15	53 ± 13	0.018*
Sex				
	Female	53.9	42.4	0.04
	Male	46.1	57.6	
BMI		32.24 ± 8.48	32.81 ± 8.46	0.549
SAPS 3		59 ± 15	58 ± 13	0.698
Comorbidities				
	Cardiopathy	67.5	52.2	0.006
	Pneumopathy	21.1	21,1	0,913
	Nephropathy	9.8	8,1	0,597
	Endocrinopathy	67.6	64,3	0,535
	Neoplasia	4.9	3,7	0,568
	Others	40.2	32	0,125
Corticosteroid therapy		100	97,5	0,218†
Preextubation tracheostomy		3.9	17,4	0,002
Tracheostomy during hospitalization		41.2	20,2	< 0,001
Initial CRP		206.4 ± 106.3	189,2 ± 99,9	0,142
Initial lymphocyte count		829 ± 421	866 ± 588	0,702
Initial NLR (n/uL)		11.2 [7.7 – 18.8]	12,4 [8 – 18,5]	0,713
CRP variation (%)		-48.28 [-71.84 - -17.31]	-70,35 [-84,93 - -43,03]	<0,001*
Lymphocyte count variation (%)		+33.69 [-9.09 – 107.06]	+68,55 [10,99 – 153,85]	< 0,001*
NLR variation (%)		-20.80 [-55.31 – 21.75]	-40,04 [-66,19 - −6,25]	< 0,001*
Time on MV until extubation (days)		11 [7 – 16]	11 [6 – 20]	0,001*
Time in ICU (days)		25 [18 – 36]	16 [10 – 26]	< 0,001*
Hospitalization time (days)		38 [25 - 50]	28 [18 – 43]	0.0004*
ICU outcome				
	Discharge	67.5	96.3	< 0.001
	Death	12.7	2.0	
	Hospital transfer	19.6	1.7	
Hospital outcome				
	Discharge	61.8	84.8	< 0.001
	Death	15.7	4.2	
	Hospital transfer	22.5	11	

BMI - body mass index; SAPS - Simplified Acute Physiology Score; CRP - C-reactive protein; NLR - neutrophil-lymphocyte ratio; MV - mechanical ventilation; ICU - intensive care unit.

* Analysis with spline transformation;

† Fisher's exact test used. The results expressed as mean ± standard deviation, % median [interquartile range].

## RESULTS

**Figure 1:**
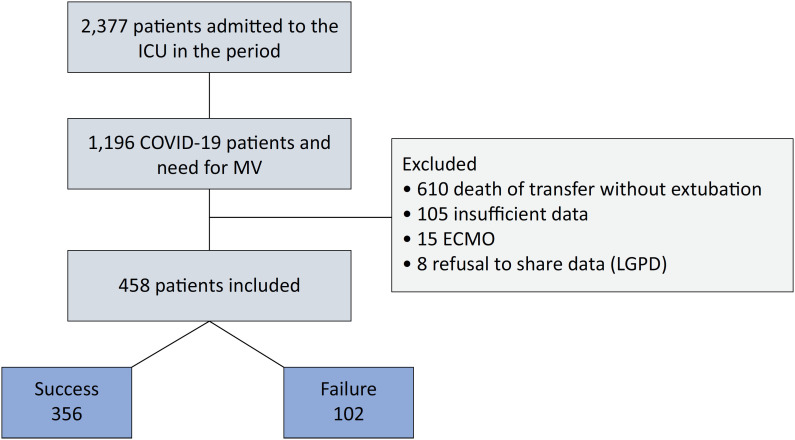
Patient flowchart. ICU - intensive care unit; ECMO - extracorporeal membrane oxygenation; LGPD - General Data Protection Law (*Lei Geral de Proteção de Dados*).

Between March 2020 and April 2021, 2,377 patients were admitted to the ICU of *Hospital de Clínicas de Porto Alegre*, of which 1,196 were eligible for the study. The 1,181 noneligible patients were non-COVID-19 patients or COVID-19 patients without the need for MV. Of the total eligible patients, 458 were included in the analysis, with 356 in the Successful Weaning Group and 102 in the Failure Weaning Group. The others were excluded as described in figure 1.

### Sample characteristics

Table 1 shows the cohort's profile. The Extubation Failure Group had more patients with heart disease (67.5% *versus* 52.2%; p = 0.006) and a greater need for tracheostomy during hospitalization (41.2% *versus* 20.2%; p < 0.001). Among patients who were successfully weaned, there were more tracheostomies performed before weaning (3.9% *versus* 17.4%; p = 0.002), as well as a significant reduction in the serum levels of CRP and NLR and an increase in total lymphocytes (-70.35% *versus* -48.28%; p < 0.001; -40.04% *versus* −20.8%; p < 0.001; +68.55% *versus* +33.69%; p < 0.001). Other characteristics were similar across the groups.

### Primary outcomes

**Figure 2:**
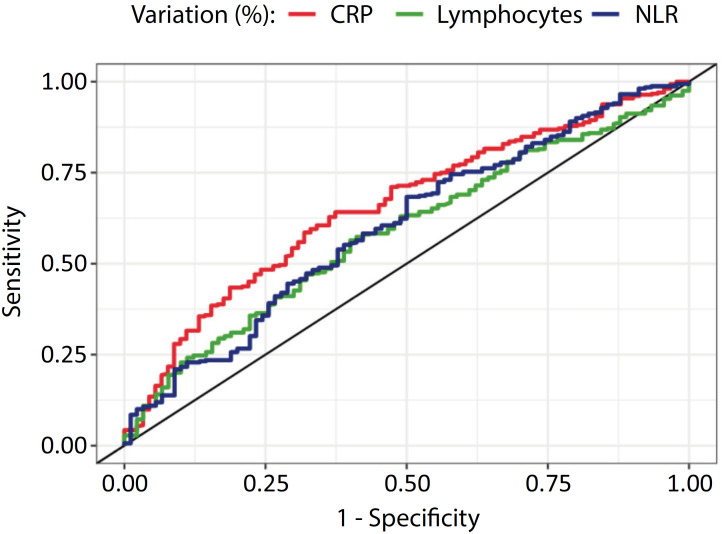
ROC curves: biomarkers and extubation success. CRP - C-reactive protein; NLR - neutrophil-lymphocyte ratio.

The cutoff points found from the ROC curves with the best sensitivity and specificity for successful extubation were −62.4% for CRP, +45.7% for total lymphocytes, and −32.9% for the NLR (Table 2 and Figure 2). These points were significantly associated with greater success in extubation. In the multivariable analysis and after adjusting for heart disease, preextubation ventilation time, and preextubation tracheostomy, only the variation in CRP remained statistically significant (OR 2.6; 95%CI 1.51 – 4.5; p < 0.001), as described in table 3. The analysis of combined biomarkers (CRP and lymphocytes, CRP and NLR, lymphocytes and NLR, CRP and lymphocytes and NLR) did not add sensitivity or specificity to the analysis of CRP kinetics alone (Supplementary Material).

**Table 2:** ROC curves: biomarkers and successful extubation

Indicators	AUC	95%CI	Cutoff point (%)	Sensitivity (%)	Specificity (%)
CRP	0.653	0.59 - 0.71	-62.4	62.8	63.3
Lymphocytes	0.585	0.52 - 0.64	+45.7	57.9	57.8
NLR	0.60	0.53 - 0.67	-32.9	58.3	57.8

AUC - area under the curve; 95%CI - 95% confidence interval CRP - C-reactive protein; NLR - neutrophil-to-lymphocyte ratio.

**Table 3:** Logistic regression: biomarker analysis and extubation success

	Univariate analysis OR (95%CI)	Multivariate analysis OR (95%CI)*
CRP variation −62.4%	2.91 (1.82 – 4.83)	2.6 (1.51 – 4.5)
Lymphocytes variation +45.7%	1.82 (1.14 – 2.93)	1.38 (0.75 – 2.54)
NLR variation −32.9%	1.91 (1.19 – 3.07)	1.33 (0.72 – 2.46)

OR - odds ratio; 95%CI - 95% confidence interval; CRP - C-reactive protein; NLR - neutrophil-lymphocyte ratio.

* Multivariate analysis - adjusted for heart disease, preextubation tracheostomy and time of mechanical ventilation until extubation.

The sensitivity and specificity values found for a −62.4% reduction in CRP were 62.8% and 63.3%, respectively. When CRP maintains its values without alteration, the found sensitivity is 89%. However, a reduction of -83.1% yields a specificity of 90%. For a 45.7% increase in lymphocytes, the sensitivity and specificity were 57.9% and 57.8%, respectively. For a −32.9% reduction in NLR, they were 58.3% and 57.8%, respectively. The positive likelihood ratio for CRP was 1.71, and the negative likelihood ratio was 0.58, based on the outcome of successful extubation.

### Secondary outcomes

The length of stay in the ICU and hospital was significantly longer in the Extubation Failure Group (25 [18 – 36) *versus* 16 [10 – 26]; p < 0.001 and 38 [25 - 50] *versus* 28 (18 – 43); p = 0.0004). The ICU and hospital discharge rates were significantly higher in successful patients (67.5% *versus* 96.3%; p < 0.001; 61.8% *versus* 84.8%; p < 0.001). The hospital mortality rate in patients with failure was 15.7% compared to 4.2% in the successful group.

## DISCUSSION

In this retrospective cohort of COVID-19 patients on mechanical ventilation, an association was demonstrated between a decrease in serum levels of CRP and successful weaning from ventilation. Our study suggests that the decrease in CRP levels in SARS-CoV-2 patients on MV is an indicator of resolution of COVID infection, aiding in identifying the point at which a patient is more likely to succeed once they meet the already validated readiness criteria.

As in our study, data in the literature support the use of CRP as a relevant biomarker in COVID-19 patients, CRP is strongly correlated with disease progression and is an independent predictor of severity, as well as the need for mechanical ventilation.^(27-31)^ More relevant than isolated measurements, the kinetics of CRP seem to be the main point for clinical practice, as demonstrated in our study. Prepandemic studies in patients with bacterial infections have already analyzed the kinetics of CRP in survivors and nonsurvivors.^(32-34)^ Apparently, persistently high or increasing levels of CRP suggest the maintenance of inflammatory activity or are associated with worse prognosis, while their reduction indicates resolution of the inflammatory process and better outcome.^(12)^

Such studies assess the kinetics of CRP with prognostic outcomes but without an analysis of ventilatory weaning. Other studies, such as Forgiarini et al., have examined inflammatory factors in the ventilatory weaning of non-COVID-19 patients. However, the kinetics of CRP were not evaluated, and only absolute values were evaluated.^(6)^ Although widely studied in bacterial infections, the literature on viral infections such as SARS-CoV-2 is scarce. Thus, this is the first study to relate the kinetics of inflammatory markers to successful extubation.

However, the decrease in CRP levels should not be a variable that, alone, identifies COVID-19 patients suitable for weaning. In our study, although there was an association between CRP variation and extubation success, the area under the ROC curve demonstrated poor accuracy in discriminating the extubation outcome, with low sensitivity and specificity. Our study indicates that this biomarker can, together with other variables associated with success and readiness for extubation, help identify patients in their best condition for weaning from ventilation. This hypothesis needs to be tested in clinical trials.

Among our findings, the association between lymphocyte count or NLR and weaning was not confirmed after multivariate analysis. In the literature, articles suggest a lower prognostic accuracy of lymphopenia and an increased NLR in relation to CRP in distinguishing disease severity.^(35)^ The accuracy of both hematological parameters seems to be impaired once their counts are influenced by factors such as infections by other pathogens or medications, such as glucocorticoids, which are the standard treatment for SARS-CoV-2 and were used in 98% of our population.^(36)^

Another finding of our study, as a secondary outcome, is the shorter ICU and hospital stays in patients with successful extubation. In addition, ICU and hospital mortality were higher in patients with extubation failure. There is evidence in the literature that failure is associated with an increased mortality rate, either due to the selection of higher risk patients or associated deleterious effects such as bronchoaspiration, pneumonia, and atelectasis.^(5)^ Such a causal relationship cannot be affirmed from this observational study design.

### Limitations

Our study has some limitations. It is a single-center study, so these findings should be replicated in other centers to confirm the hypotheses generated. Due to its observational nature, confounding factors may influence its outcome. Appropriate statistical analyses were employed to correct these factors. One unmeasured factor in this study and a possible confounder is the presence of associated bacterial infection, which can impact the duration of ventilation, mortality, and weaning success. However, a systematic review showed a low prevalence of bacterial infections associated with COVID-19 patients, regardless of whether the bacterial infections were secondary infections (13.5%) or coinfections (7.0%). This review points to a large dissociation between evidence of bacterial infection and the use of antimicrobials, as only 16% of studies reported indications of bacterial infection, while 54% of patients received empirical antibiotic therapy.^(37)^ Furthermore, it should be mentioned that critically ill COVID-19 patients admitted to the ICU may have higher rates of secondary bacterial infection, reaching more than 50% of cases.^(38)^

Another limitation is the nonmeasurement of other variables related to weaning success, such as the weaning readiness criteria and the type of spontaneous ventilation test used. In addition, survival bias may be present, as the number of exclusions due to death was high due to the global severity of the disease.

## CONCLUSION

In this study, the decrease in C-reactive protein levels was significantly associated with successful extubation in mechanically ventilated COVID-19 patients, suggesting that this biomarker may contribute to decision-making in this context. Total lymphocytes and the neutrophil-to-lymphocyte ratio did not maintain their association after multivariate analysis.
