# Supplementary material for: Association of biomarkers with successful ventilatory weaning in COVID-19 patients: an observational study
Source: Crit Care Sci. 2024 Mar 25;36:e20240158en. doi: 10.62675/2965-2774.20240158-en (PMC11098064; doi:10.62675/2965-2774.20240158-en)
Supplement: Translation

# Translation: Associação entre biomarcadores e sucesso do desmame ventilatório em pacientes com COVID-19: um estudo observacional

**DOI:** 10.62675/2965-2774.20240158-pt

**Authors:** Bruna Schneider, Raquel Almeida de Oliveira, Gilberto Friedman, Rafael Barberena Moraes

**Affiliations:** 1 Porto Alegre RS Brasil Programa de Pós-Graduação em Ciências Pneumológicas, Universidade Federal do Rio Grande do Sul - Porto Alegre (RS), Brasil.; 2 Porto Alegre RS Brasil Universidade Federal do Rio Grande do Sul - Porto Alegre (RS), Brasil.

**Keywords:** Biomarcadores, COVID-19, Infecções por coronavírus, Extubação, Intubação intratraqueal, Respiração artificial, Desmame do respirador, Unidades de terapia intensiva

## Abstract

**Objetivo:**

Avaliar a associação de biomarcadores com o sucesso do desmame ventilatório em pacientes com COVID-19.

**Métodos:**

Trata-se de estudo observacional, retrospectivo e de centro único realizado entre março de 2020 e abril de 2021. Foram avaliados a proteína C-reativa, os linfócitos totais e a relação neutrófilos/linfócitos durante o atrito e a extubação; mediu-se a variação desses valores de biomarcadores. O desfecho primário foi o sucesso da extubação. As curvas ROC foram desenhadas para encontrar os melhores pontos de corte dos biomarcadores segundo a sensibilidade e a especificidade. A análise estatística foi realizada por meio de regressão logística.

**Resultados:**

Dos 2.377 pacientes admitidos na unidade de terapia intensiva, 458 foram incluídos na análise, 356 no Grupo Sucesso do desmame e 102 no Grupo Fracasso do desmame. Os pontos de corte encontrados nas curvas ROC foram −62,4% para proteína C-reativa, +45,7% para linfócitos totais e −32,9% para relação neutrófilo/linfócito. Esses pontos foram significativamente associados ao maior sucesso da extubação. Na análise multivariada, apenas a variação da proteína C-reativa permaneceu estatisticamente significativa (RC 2,6; IC95% 1,51 – 4,5; p < 0,001).

**Conclusão:**

Neste estudo, uma diminuição nos níveis de proteína C-reativa foi associada ao sucesso da extubação em pacientes com COVID-19. Os linfócitos totais e a relação neutrófilos/linfócitos não mantiveram a associação após a análise multivariada. No entanto, uma diminuição nos níveis de proteína C-reativa não deve ser usada como única variável para identificar pacientes com COVID-19 adequados para o desmame; como em nosso estudo, a área sob a curva ROC demonstrou baixa precisão na discriminação dos resultados de extubação, com baixas sensibilidade e especificidade.

## INTRODUÇÃO

O desmame ventilatório é um desafio constante para os intensivistas e se tornou ainda mais complexo durante a pandemia do coronavírus da síndrome respiratória aguda grave 2 (SARS-CoV-2). Os fatores enfrentados são a hipoxemia grave, o uso de corticosteroides, a ventilação mecânica (VM) prolongada, a sedação e a imobilização por longos períodos, o aumento da demanda ventilatória, o curso variável da doença, a sobrecarga de trabalho e a falta de recursos.^(1-4)^

Apesar da adoção de protocolos e da adesão às recomendações das diretrizes, as taxas de fracasso de extubação permanecem em torno de 15 a 30%.^(5,6)^ Em pacientes com doença causada pela doença pelo coronavírus 2019 (COVID-19), essa taxa é aproximadamente três vezes maior do que em pacientes sem COVID-19.^(7)^ O fracasso da extubação está associado ao aumento da mortalidade hospitalar, ao prolongamento da internação na unidade de terapia intensiva (UTI) e ao aumento da necessidade de traqueostomia.^(8,9)^ Entretanto, o prolongamento desnecessário da VM também está associado a um maior risco de infecção e mortalidade na UTI.^(5,10,11)^ Nesse contexto, torna-se necessária a tentativa de identificar fatores que auxiliem na tomada de decisão quanto ao momento ideal para a interrupção da VM.

Apesar de sua sensibilidade e especificidade limitadas, biomarcadores como a proteína C-reativa (PCR), a procalcitonina (PCT) e a relação neutrófilos/linfócitos (RNL) têm sido amplamente utilizados em infecções bacterianas, desempenhando um papel diagnóstico, prognóstico, de estratificação de risco e definidor da duração do antibiótico.^(12-16)^ Os linfócitos também desempenham um papel crucial na resposta inflamatória e no equilíbrio do sistema imunológico, e a linfopenia, um achado comum na infecção por SARS-CoV-2, está significativamente associada a desfechos desfavoráveis.^(17-19)^ Em geral, à medida em que a doença inflamatória progride, a contagem de linfócitos diminui, e a contagem de neutrófilos aumenta. A RNL, ferramenta facilmente calculada a partir do hemograma completo, é um indicador de inflamação sistêmica e um fator de risco independente para um prognóstico desfavorável.^(20,21)^ Até o momento, poucos estudos avaliaram a associação entre esses biomarcadores em pacientes com COVID-19 e desfechos clínicos desfavoráveis, mas nenhum avaliou sua relação com o desmame.^(18,22-24)^

O objetivo deste estudo foi avaliar a associação dos biomarcadores PCR, linfócitos totais e RNL com o sucesso da extubação em pacientes com insuficiência ventilatória secundária à SARS-CoV-2.

## MÉTODOS

### Desenho e população do estudo

Trata-se de um estudo retrospectivo, observacional e unicêntrico realizado em pacientes internados na UTI do Hospital de Clínicas de Porto Alegre entre março de 2020 e abril de 2021. Os pacientes incluídos no estudo atenderam aos seguintes critérios: diagnóstico confirmado de infecção por SARS-CoV-2 por meio de biologia molecular ou teste rápido de antígeno, idade igual ou superior a 18 anos e submetidos a VM para síndrome respiratória aguda grave. Os critérios de exclusão incluíram infecção por SARS-CoV-2 ocorrida após a necessidade de VM, uso de oxigenação por membrana extracorpórea (ECMO), óbito ou transferência para outro hospital sem que a extubação fosse realizada, uso de inibidores de interleucina 6, ausência de coleta dos biomarcadores analisados e recusa de compartilhamento de dados por meio da Lei Geral de Proteção de Dados (LGPD), em vigor no Brasil.

O estudo foi aprovado pelo Comitê de Ética e Pesquisa (CEP) da instituição (CAAE 40843120.4.0000.5327). Os autores assinaram um Termo de Compromisso de Utilização de Dados, confirmando seu compromisso com o uso anônimo dos dados. Devido ao anonimato dos pacientes e à ausência de intervenções diagnósticas ou terapêuticas, dispensou-se o Termo de Consentimento Livre e Esclarecido. Os resultados foram relatados conforme as diretrizes *Strengthening the Reporting of Observational Studies in Epidemiology* (STROBE).

### Coleta de dados

Os seguintes dados foram coletados por meio de revisão de prontuários: características gerais do paciente, como sexo, idade, comorbidades, índice de massa corporal (IMC) e *Simplified Acute Physiology Score 3* (SAPS 3); data de admissão, alta hospitalar ou óbito; data de intubação e extubação; uso de corticosteroides; realização de traqueostomia; e biomarcadores (PCR, linfócitos totais e RNL) coletados até 24 horas antes ou depois do momento da intubação e extubação. A partir desses dados laboratoriais, foram calculadas as variações nos níveis desses biomarcadores entre a data da intubação e a data da primeira extubação. Os possíveis fatores de confusão foram selecionados, *a priori*, com base no conhecimento clínico e na literatura existente.

### Desfechos e definições

Neste estudo, comparamos a cinética dos biomarcadores entre os Grupos Fracasso e Sucesso da Extubação. O Grupo Fracasso foi considerado a necessidade de retorno à VM em menos de 48 horas.

Como desfechos secundários, avaliamos o tempo de VM até a extubação, o tempo de internação hospitalar, a mortalidade na UTI, a mortalidade hospitalar e o uso de traqueostomia. Os critérios para extubação foram avaliados e decididos pela equipe à beira do leito, seguindo os protocolos institucionais, sem a influência dos pesquisadores. Os resultados de transferência hospitalar foram incluídos devido à pandemia, com pacientes sendo transferidos para hospitais de menor complexidade devido à alta demanda de recursos. O parâmetro "traqueostomia pré-extubação" refere-se à realização de uma traqueostomia durante a mesma hospitalização sem extubação prévia. Essa prática é incomum fora do contexto de uma pandemia; no entanto, durante a pandemia, ela foi empregada em alguns pacientes com desmame difícil e prolongado, pelo menos em nosso centro. Nesses pacientes traqueostomizados, o sucesso da extubação foi considerado a manutenção da independência da VM por pelo menos 48 horas.

### Análise estatística

A amostra foi considerada a partir da lista total de hospitalizações no período. As variáveis categóricas foram relatadas como porcentagem, e as variáveis contínuas foram relatadas como média ± desvio-padrão (DP) ou mediana (intervalo interquartil [IQ]). As curvas de *receiver operating characteristic* (ROC) foram desenhadas para encontrar o melhor ponto de corte dos biomarcadores em função da melhor sensibilidade e especificidade. As associações foram feitas com o modelo de regressão logística, exceto para a variável "uso de corticosteroides", na qual foi usado o teste exato de Fisher, uma vez que, em um dos grupos, nenhum paciente usou corticosteroides. Foi detectada uma associação não linear envolvendo idade, tempo de VM até a extubação, tempo de internação na UTI e tempo de hospitalização com sucesso da extubação, tendo sido aplicada uma transformação *spline*.

Nas análises multivariáveis, foram usadas as seguintes variáveis de ajuste: doença cardíaca prévia, traqueostomia pré-extubação e tempo de VM até a extubação, definidas com base na significância estatística, conforme descrito na tabela 1 (p < 0,05). As outras variáveis com significância na tabela 1 não foram usadas devido à colinearidade com as já mencionadas. Os resultados das análises uni e multivariáveis foram expressos em razões de chances (RCs) e respectivos intervalos de confiança de 95% (IC95%). Em todas as análises, adotou-se valor de p < 0,05 como nível de significância estatística. O *software* utilizado foi o PASW Statistics for Windows versão 18.0 e o R versão 4.2.0.^(25,26)^

**Tabela 1:** Características da população

Características		Fracasso da extubação (n = 102)	Sucesso da extubação (n = 356)	Valor de p
Idade		55 ± 15	53 ± 13	0,018*
Sexo				
	Feminino	53,9	42,4	0,04
	Masculino	46,1	57,6	
IMC		32,24 ± 8,48	32,81 ± 8,46	0,549
SAPS 3		59 ± 15	58 ± 13	0,698
Comorbidades				
	Cardiopatia	67,5	52,2	0,006
	Pneumopatia	21,1	21,1	0,913
	Nefropatia	9,8	8,1	0,597
	Endocrinopatia	67,6	64,3	0,535
	Neoplasia	4,9	3,7	0,568
	Outros	40,2	32	0,125
Terapia com corticosteroides		100	97,5	0,218†
Traqueostomia pré-extubação		3,9	17,4	0,002
Traqueostomia durante a hospitalização		41,2	20,2	< 0,001
PCR inicial		206,4 ± 106,3	189,2 ± 99,9	0,142
Contagem de linfócitos inicial		829 ± 421	866 ± 588	0,702
RNL inicial (n/uL)		11,2 [7,7 – 18,8]	12,4 [8 – 18,5]	0,713
Variação da PCR (%)		-48,28 [-71,84 - -17,31]	-70,35 [-84,93 - -43,03]	< 0,001*
Variação da contagem de linfócitos (%)		+33,69 [-9,09 – 107,06]	+68,55 [10,99 – 153,85]	< 0,001*
Variação da RNL (%)		-20,80 [-55,31 – 21,75]	-40,04 [-66,19 - −6,25]	< 0,001*
Duração da VM até a extubação (dias)		11 [7 – 16]	11 [6 – 20]	0,001*
Duração da internação na UTI (dias)		25 [18 – 36]	16 [10 – 26]	< 0,001*
Duração da hospitalização (dias)		38 [25 - 50]	28 [18 – 43]	0,0004*
Desfecho na UTI				
	Alta hospitalar	67,5	96,3	< 0,001
	Morte	12,7	2,0	
	Transferência hospitalar	19,6	1,7	
Desfecho hospitalar				
	Alta hospitalar	61,8	84,8	< 0,001
	Morte	15,7	4,2	
	Transferência hospitalar	22,5	11	

IMC - índice de massa corporal; SAPS - *Simplified Acute Physiology Score*; PCR - proteína C-reativa; RNL - relação neutrófilos-linfócitos; VM - ventilação mecânica; UTI - unidade de terapia intensiva.

* Análise com transformação *spline*;

† aplicação do teste exato de Fisher. Resutlados expressos por média ± desvio-padrão, %, n ou mediana [intervalo interquartil].

## RESULTADOS

Entre março de 2020 e abril de 2021, 2.377 pacientes foram internados na UTI do Hospital de Clínicas de Porto Alegre, dos quais 1.196 eram candidatos ao estudo. Os 1.181 pacientes não selecionados eram pacientes sem COVID-19 ou pacientes com COVID-19 sem necessidade de VM. Do total de pacientes aptos, 458 foram incluídos na análise, sendo 356 no Grupo Sucesso do Desmame e 102 no Grupo Fracasso do desmame. Os demais foram excluídos conforme descrito na figura 1.

**Figura 1:** Fluxograma do paciente.

UTI - unidade de terapia intensiva; ECMO - oxigenação por membrana extracorpórea; LGPD - Lei Geral de Proteção de Dados.

### Características da amostra

A tabela 1 mostra o perfil da coorte. O Grupo Fracasso da extubação tinha mais pacientes com doença cardíaca (67,5% *versus* 52,2%; p = 0,006) e mais necessidade de traqueostomia durante a hospitalização (41,2% *versus* 20,2%; p < 0,001). Entre os pacientes que foram desmamados com sucesso, houve mais traqueostomias realizadas antes do desmame (3,9% *versus* 17,4%; p = 0,002), bem como uma redução significativa nos níveis séricos de PCR e RNL e um aumento no total de linfócitos (-70,35% *versus* -48,28%; p < 0,001; -40,04% *versus* −20,8%; p < 0,001; +68,55% *versus* +33,69%; p < 0,001). Outras características foram semelhantes entre os grupos.

### Desfechos primários

Os pontos de corte encontrados nas curvas ROC com a melhor sensibilidade e especificidade para sucesso na extubação foram −62,4% para PCR, +45,7% para linfócitos totais e −32,9% para RNL (Tabela 2 e Figura 2). Esses pontos foram significativamente associados a maior sucesso na extubação. Na análise multivariável e após o ajuste para doença cardíaca, tempo de ventilação pré-extubação e traqueostomia pré-extubação, somente a variação da PCR permaneceu estatisticamente significativa (RC 2,6; IC95% 1,51 – 4,5; p < 0,001), conforme descrito na tabela 3. A análise de biomarcadores combinados (PCR e linfócitos, PCR e RNL, linfócitos e RNL, PCR e linfócitos e RNL) não adicionou sensibilidade ou especificidade à análise da cinética da PCR isoladamente (Material Suplementar).

**Tabela 2:** Curvas ROC: biomarcadores e sucesso da extubação

Indicadores	ASC	IC95%	Ponto de corte (%)	Sensibilidade (%)	Especificidade (%)
PCR	0,653	0,59 - 0,71	-62,4	62,8	63,3
Linfócitos	0,585	0,52 - 0,64	+45,7	57,9	57,8
RNL	0,60	0,53 - 0,67	-32,9	58,3	57,8

ASC - área sob a curva; IC95% - intervalo de confiança de 95%; PCR - proteína C-reativa; RNL - relação neutrófilos/linfócitos.

**Figura 2:** Curvas ROC: biomarcadores e sucesso da extubação.

PCR - proteína C-reativa; RNL - relação neutrófilo-linfócito.

**Tabela 3:** Regressão logística: análise de biomarcadores e sucesso da extubação

	Análise univariada RC (IC95%)	Análise multivariada RC (IC95%)*
Variação da PCR −62,4%	2,91 (1,82 – 4,83)	2,6 (1,51 – 4,5)
Variação de linfócitos +45,7%	1,82 (1,14 – 2,93)	1,38 (0,75 – 2,54)
Variação da RNL −32,9%	1,91 (1,19 – 3,07)	1,33 (0,72 – 2,46)

RC - razão de chances; IC95% - intervalo de confiança de 95%; PCR - proteína C-reativa; RNL - razão neutrófilos-linfócitos.

* Análise multivariada ajustada para doença cardíaca, traqueostomia pré-extubação e tempo de ventilação mecânica até a extubação.

Os valores de sensibilidade e especificidade encontrados para uma redução de −62,4% na PCR foram 62,8 e 63,3%, respectivamente. Quando a PCR manteve seus valores inalterados, a sensibilidade encontrada foi de 89%. Entretanto, uma redução de -83,1% produziu uma especificidade de 90%. Para um aumento de 45,7% nos linfócitos, a sensibilidade e a especificidade foram de 57,9 e 57,8%, respectivamente. Para uma redução de −32,9% na RNL, elas foram de 58,3 e 57,8%, respectivamente. A razão de probabilidade positiva da PCR foi de 1,71, e a razão de probabilidade negativa foi de 0,58, com base no desfecho de sucesso da extubação.

### Desfechos secundários

O tempo de internação na UTI e no hospital foi significativamente maior no Grupo Fracasso da extubação (25 [18 – 36] *versus* 16 [10 – 26]; p < 0,001 e 38 [25 - 50] *versus* 28 [18 – 43]; p = 0,0004). A taxa de mortalidade hospitalar em pacientes com fracasso foi de 15,7% em comparação com 4,2% no grupo com sucesso.

## DISCUSSÃO

Nesta coorte retrospectiva de pacientes com COVID-19 em VM, foi demonstrada uma associação entre a diminuição dos níveis séricos de PCR e o sucesso do desmame da ventilação. Nosso estudo sugere que a diminuição dos níveis de PCR em pacientes com SARS-CoV-2 em VM é um indicador da resolução da infecção por COVID-19, ajudando a identificar o ponto em que um paciente tem maior probabilidade de sucesso, uma vez que atende aos critérios de prontidão já validados.

Assim como em nosso estudo, os dados da literatura sustentam o uso da PCR como um biomarcador relevante em pacientes com COVID-19. A PCR está fortemente correlacionada com a progressão da doença e é um preditor independente de gravidade e da necessidade de VM.^(27-31)^ Mais relevante do que medições isoladas, a cinética da PCR parece ser o ponto principal para a prática clínica, conforme demonstrado em nosso estudo. Estudos pré-pandêmicos em pacientes com infecções bacterianas já analisaram a cinética da PCR em sobreviventes e não sobreviventes.^(32-34)^ Aparentemente, níveis persistentemente altos ou crescentes de PCR sugerem a manutenção da atividade inflamatória ou estão associados a prognóstico desfavorável, enquanto sua redução indica resolução do processo inflamatório e desfecho favorável.^(12)^

Esses estudos avaliam a cinética da PCR com resultados prognósticos, mas sem uma análise do desmame ventilatório. Outros estudos, como o de Forgiarini et al., analisaram fatores inflamatórios no desmame ventilatório de pacientes sem COVID-19. No entanto, a cinética da PCR não foi avaliada, e apenas os valores absolutos foram observados.^(6)^ Embora amplamente estudada em infecções bacterianas, há escassez de literatura sobre infecções virais como a SARS-CoV-2. Portanto, este é o primeiro estudo a relacionar a cinética dos marcadores inflamatórios ao sucesso da extubação.

No entanto, a diminuição dos níveis de PCR não deve ser uma variável que, isoladamente, identifique pacientes com COVID-19 aptos ao desmame. Em nosso estudo, embora tenha havido uma associação entre a variação da PCR e o sucesso da extubação, a área sob a curva ROC demonstrou baixa precisão na discriminação do desfecho da extubação, com baixa sensibilidade e especificidade. Nosso estudo indica que esse biomarcador pode, com outras variáveis associadas ao sucesso e à prontidão para a extubação, ajudar a identificar pacientes em suas melhores condições para o desmame da ventilação. Essa hipótese precisa ser testada em estudos clínicos.

Entre nossos achados, a associação entre a contagem de linfócitos ou a RNL e o desmame não foi confirmada após a análise multivariada. Na literatura, os artigos sugerem uma menor precisão prognóstica da linfopenia e um aumento da RNL em relação à PCR na distinção da gravidade da doença.^(35)^ A precisão de ambos os parâmetros hematológicos parece ser prejudicada uma vez que suas contagens são influenciadas por fatores como infecções por outros patógenos ou medicamentos, como os glicocorticoides, que são o tratamento padrão para a SARS-CoV-2 e foram usados em 98% da nossa população.^(36)^

Outro achado de nosso estudo, como desfecho secundário, é o menor tempo de internação na UTI e hospitalar em pacientes com sucesso da extubação. Há evidências na literatura de que o fracasso está associado a um aumento da taxa de mortalidade, seja devido à seleção de pacientes de maior risco ou a efeitos deletérios associados, como broncoaspiração, pneumonia e atelectasia.^(5)^ Essa relação causal não pode ser afirmada a partir do desenho deste estudo observacional.

### Limitações

Nosso estudo tem algumas limitações. Trata-se de um estudo unicêntrico, portanto, esses achados devem ser replicados em outros centros para confirmar as hipóteses geradas. Devido à sua natureza observacional, fatores de confusão podem influenciar seu desfecho. Análises estatísticas apropriadas foram empregadas para corrigir esses fatores. Um fator não medido neste estudo e um possível fator de confusão é a presença de infecção bacteriana associada, que pode afetar a duração da ventilação, a mortalidade e o sucesso do desmame. No entanto, uma revisão sistemática mostrou baixa prevalência de infecções bacterianas associadas a pacientes com COVID-19, independentemente de as infecções bacterianas serem secundárias (13,5%) ou coinfecções (7,0%). Essa revisão aponta para uma grande dissociação entre a evidência de infecção bacteriana e o uso de antimicrobianos, pois apenas 16% dos estudos relataram indicações de infecção bacteriana, enquanto 54% dos pacientes receberam antibioticoterapia empírica.^(37)^ Além disso, deve-se mencionar que pacientes com COVID-19 graves internados na UTI podem ter taxas mais altas de infecção bacteriana secundária, chegando a mais de 50% dos casos.^(38)^

Outra limitação é a não mensuração de outras variáveis relacionadas ao sucesso do desmame, como os critérios de prontidão para o desmame e o tipo de teste de ventilação espontânea utilizado. Além disso, o viés de sobrevida pode estar presente, pois o número de exclusões por morte foi alto devido à gravidade global da doença.

## CONCLUSÃO

Neste estudo, a diminuição dos níveis de proteína C-reativa foi significativamente associada ao sucesso da extubação em pacientes com COVID-19 ventilados mecanicamente, sugerindo que esse biomarcador pode contribuir para a tomada de decisões nesse contexto. Os linfócitos totais e a relação neutrófilos/linfócitos não mantiveram sua associação após a análise multivariada.
